# Supplementary material for: Phenylalanine impairs insulin signaling and inhibits glucose uptake through modification of IRβ
Source: Nat Commun. 2022 Jul 25;13:4291. doi: 10.1038/s41467-022-32000-0 (PMC9314339; doi:10.1038/s41467-022-32000-0)
Supplement: Supplementary file 1 — Supplementary Information [file 41467_2022_32000_MOESM1_ESM.pdf]

SUPPLEMENTARY INFORMATION

Phenylalanine Impairs Insulin Signaling and Inhibits Glucose Uptake Through Modification of IRβ

Qian Zhou, [1, 2, 3, #] Wan-Wan Sun, [3, #] Jia-Cong Chen, [1, 2] Hui-Lu Zhang, [3] Jie Liu, [1, 3] Yan Lin, [1, 2] Peng-Cheng Lin, [4] Bai-Xing Wu, [5] Yan-Peng An, [1] Lin-Huang, [1] Wen-Xing Sun, [6] Xin-Wen Zhou, [1] Yi-Ming Li, [3] Yi-Yuan Yuan, [1, 2] Jian-Yuan Zhao, [1, 2] Wei Xu, [1, 2, 3, *] and Shi-Min Zhao [1, 2, 4, *]

[1] Obstetrics & Gynecology Hospital of Fudan University, State Key Laboratory of Genetic Engineering, School of Life Sciences and Institutes of Biomedical Sciences,

[2] NHC Key Lab of Reproduction Regulation (Shanghai Institute of Planned Parenthood Research), Institute of Metabolism and Integrative Biology, Shanghai Key Laboratory of Metabolic Remodeling, and Children's Hospital of Fudan University,

[3] Endocrinology department, Huashan Hospital, 5th affiliated Hospital, Fudan University Shanghai Cancer Center,

Fudan University, Shanghai 200438, P.R. China

[4] Key Laboratory for Tibet Plateau Phytochemistry of Qinghai Province, College of Pharmacy, Qinghai University for Nationalities, Xining 810007, P. R. China

[5] Guangdong Provincial Key Laboratory of Malignant Tumor Epigenetics and Gene Regulation, Guangdong-Hong Kong Joint Laboratory for RNA Medicine, RNA Biomedical Institute, Medical Research Center, Sun Yat-Sen Memorial Hospital, Sun Yat-Sen University, Guangzhou, China 510120.

[6] Department of Nutrition and Food Hygiene, School of Public Health, Nantong University, Nantong, 226019, China

[#] These authors contributed equally

[*]Correspondence should address to: Wei Xu (xuwei_0706@fudan.edu.cn) or Shi-Min Zhao (zhaosm@fudan.edu.cn)

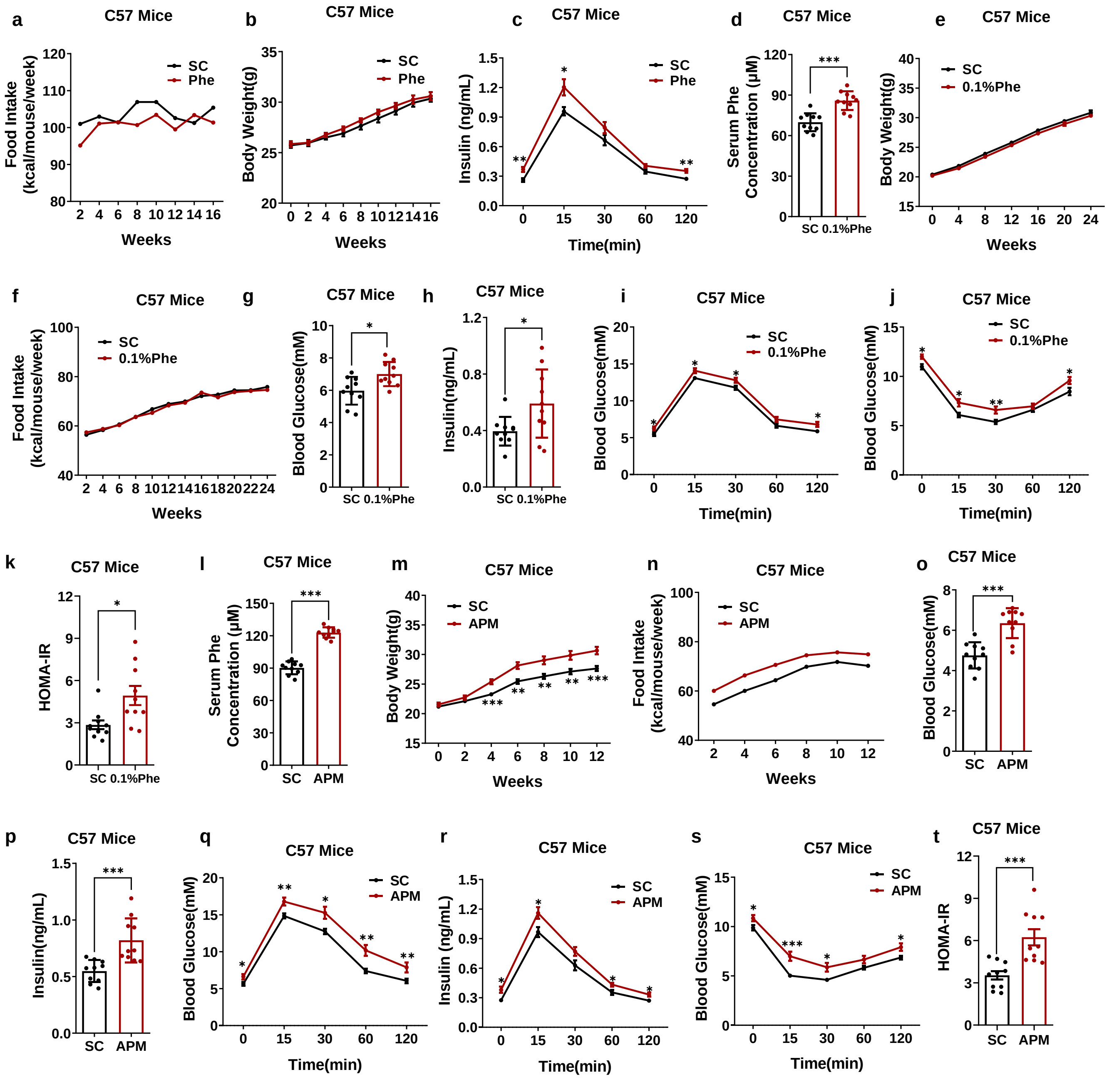

Supplementary Fig. 1, Phenylalanine- or Aspartame-supplemented chow induced symptoms of type 2 diabetes in mice, related to Fig. 1.

a-b, **Phe-chow had negligible effects on mouse food intake and body weights.** The food intake (**a**) and body weights (**b**) of SC- and Phe- chow-fed male mice were monitored during 12 weeks for Phe-chow.

c, Serum insulin concentration during GTT were monitored.

d-k, **Low dose phe-chow induced T2D symptoms in mice.** Male C57 mice (n = 10) were fed standard chow (SC) or 0.1% phenylalanine-supplemented chow (0.1%Phe), and sera Phe levels (**d**), body weights (**e**) and food intake (**f**) were monitored over time; blood glucose (**g**), serum insulin levels (**h**), glucose tolerance (**i**), insulin tolerance test (**j**), and HOMA-IR values (**k**) were measured in fasted mice after 24 weeks of feeding.

l-t, Aspartame-chow induced T2D symptoms in mice. Male C57 mice (n = 10) were fed standard chow (SC) or aspartame-supplemented chow (APM), and sera Phe levels (**l**), body weights (**m**) and food intake (**n**) were monitored over time; blood glucose (**o**), serum insulin levels (**p**), glucose tolerance (**q**) and insulin levels during glucose tolerance test (**r**), insulin tolerance test (**s**), and HOMA-IR values (**t**) were measured in fasted mice after 12 weeks of feeding.

Student's t-tests (unpaired, two-tailed) are applied for all statistical analyses in this figure. Values are expressed as the mean ± SEM. Significance was indicated as *p < 0.05, **p < 0.01, ***p < 0.001.

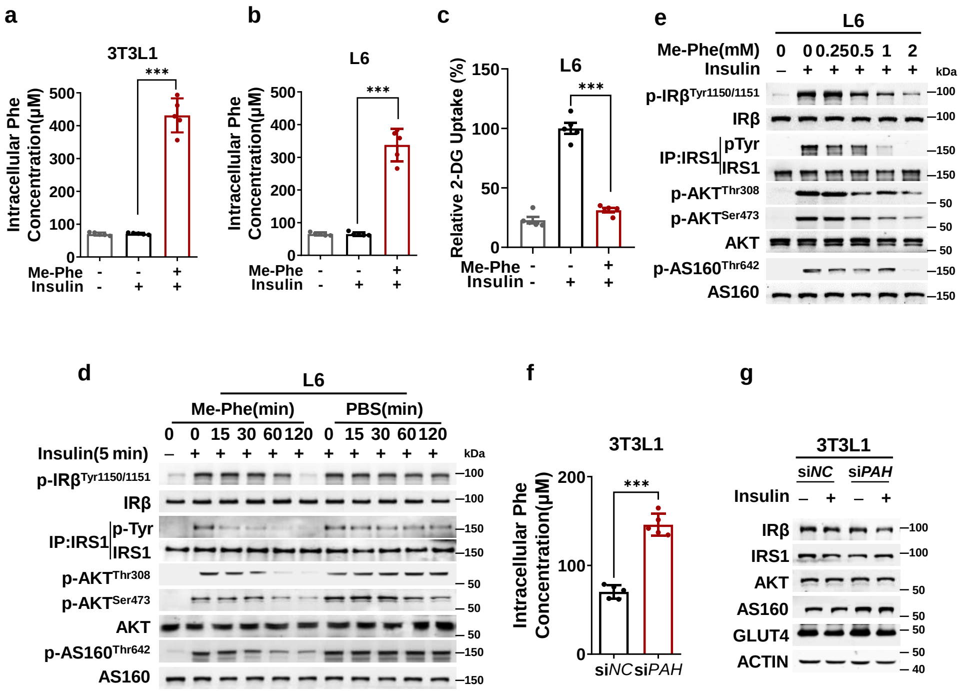

Supplementary Fig. 2, Intracellular phenylalanine blocked insulin signaling in cultured cells, related to Fig. 1.

a-b, **Me-Phe treatments elevated intracellular phenylalanine levels**. Intracellular phenylalanine concentration was measured by LC-MS for 3T3-L1 adipocytes (**a**) and L6 myocytes (**b**) that were cultured in the presence and absence of Me-Phe (n = 5).

c, **Me-Phe treatment decreased 2-DG uptake in L6 myocytes**. The 2-DG uptake of L6 myocytes was detected in the presence and absence of Me-Phe in the culture media. (n = 5).

d-e, **Me-Phe treatment impaired insulin signaling in L6 myocytes**. The time- (**d**) and dose-dependent (**e**) effects of Me-Phe on the phosphorylation of IR, IRS1, AKT and AS160 in L6 myocytes were detected.

f, PAH knockdown increased intracellular phenylalanine levels. Intracellular phenylalanine levels in 3T3-L1 adipocytes transfected with NC (scramble) or PAH siRNA were compared (n = 5).

g, PAH knockdown did not alter the protein levels of components of insulin signaling. IR, IRS1, AKT, AS160 and GLUT4 protein levels in siNC and siPAH 3T3-L1 adipocytes were detected.

Student's t-tests (unpaired, two-tailed) are applied for all statistical analyses in this figure. Values are expressed as the mean ± SEM. Significance was indicated as *$p < 0.05$, **$p < 0.01$, ***$p < 0.001$.

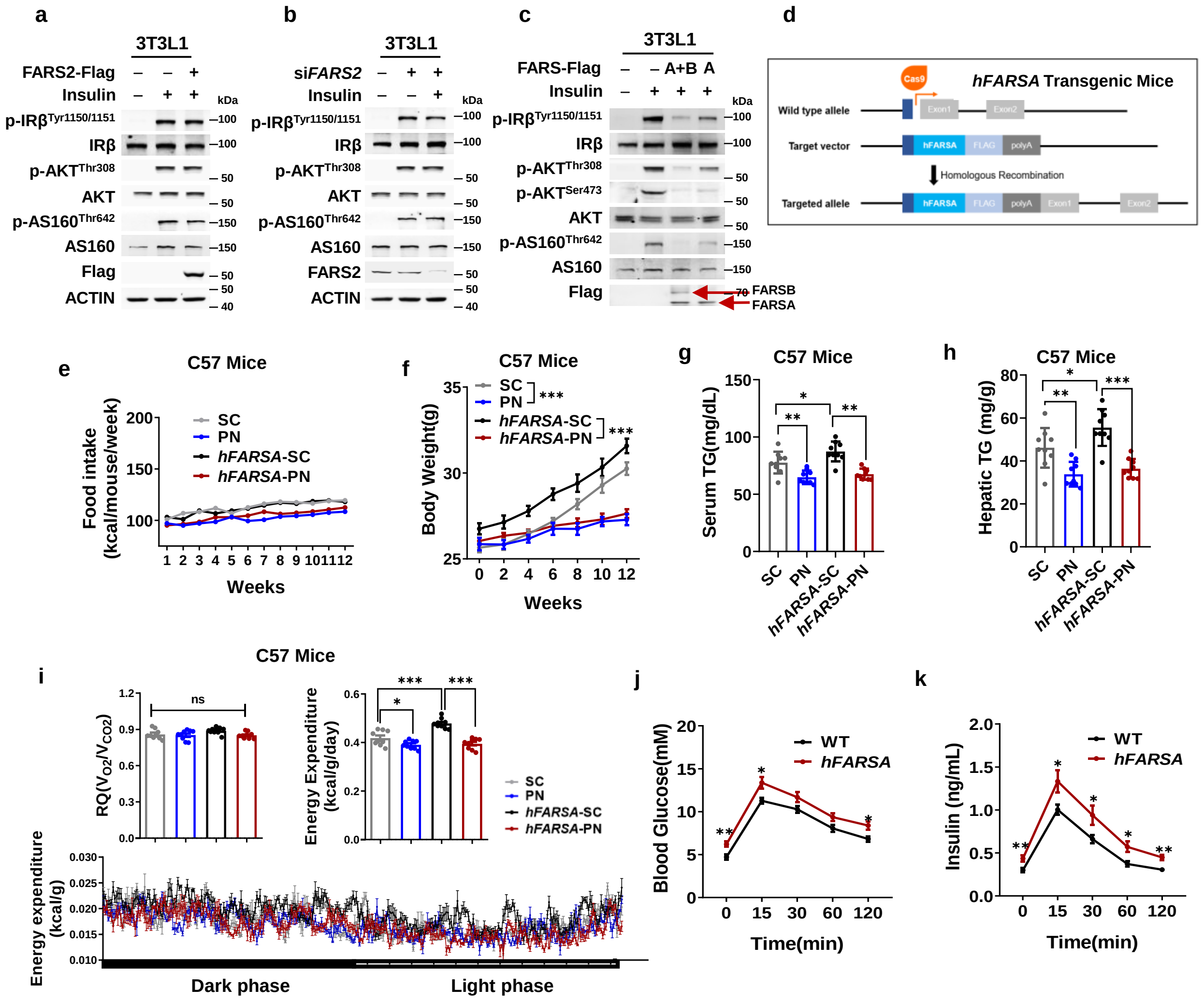

Supplementary Fig. 3, FARS blunted insulin signaling, related to Fig. 2.

a, **FARS2 overexpression did not impair insulin signaling**. Phosphorylation of IR, AKT, and AS160 in 3T3-L1 adipocytes overexpressing FARS2 was measured.

b, ***FARS2* silencing had a negligible impact on insulin signaling**. Phosphorylation of components of the insulin signaling pathway was detected in 3T3-L1 adipocytes with *FARS2* silencing.

c, **FARSA overexpression impaired insulin signaling**. The phosphorylation of insulin signaling components was detected in 3T3-L1 adipocytes that overexpressed FARSA and FARSB or FARSA only.

d, Generation of *hFARSA*-transgenic mice. A schematic diagram is shown of the strategy to generate *hFARSA*-transgenic mice.

e-i, **Physiologies of *hFARSA*-transgenic mice**. Food intake (**e**), body weights (**f**), serum TG (**g**), hepatic TG (**h**) and indirect calorimetric data (**i**) of WT and *hFARSA*-transgenic C57 mice were monitored (WT, n = 9 mice, *hFARSA*-transgenic, n= 9 mice).

j-k, ***hFARSA*-transgenic mice developed glucose intolerance**. Glucose tolerance tests (**j**) and serum insulin during GTT (**k**) were performed for fasted 8-week-old *hFARSA*-transgenic mice (WT, n = 9, *hFARSA*-transgenic, n= 10 mice).

Significance was indicated as two-tailed, unpaired, t test for j, k, one-way ANOVA test for e-i. Values are expressed as the mean ± SEM. $^*p < 0.05$, $^{**}p < 0.01$, $^{***}p < 0.001$.

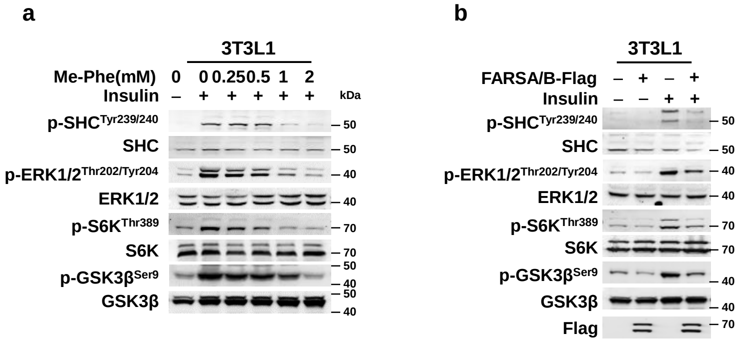

Supplementary Fig. 4, Me-Phe treatment and FARSA/B overexpression inhibited the phosphorylation of non-canonical IR targets, related to Fig. 3.

a-b, The phosphorylation of SHC, ERK1/2, S6K and GSK3β in 3T3-L1 adipocytes and Me-Phe-treated (**a**) or FARSA/B-overexpressing (**b**) 3T3-L1 adipocytes was detected.

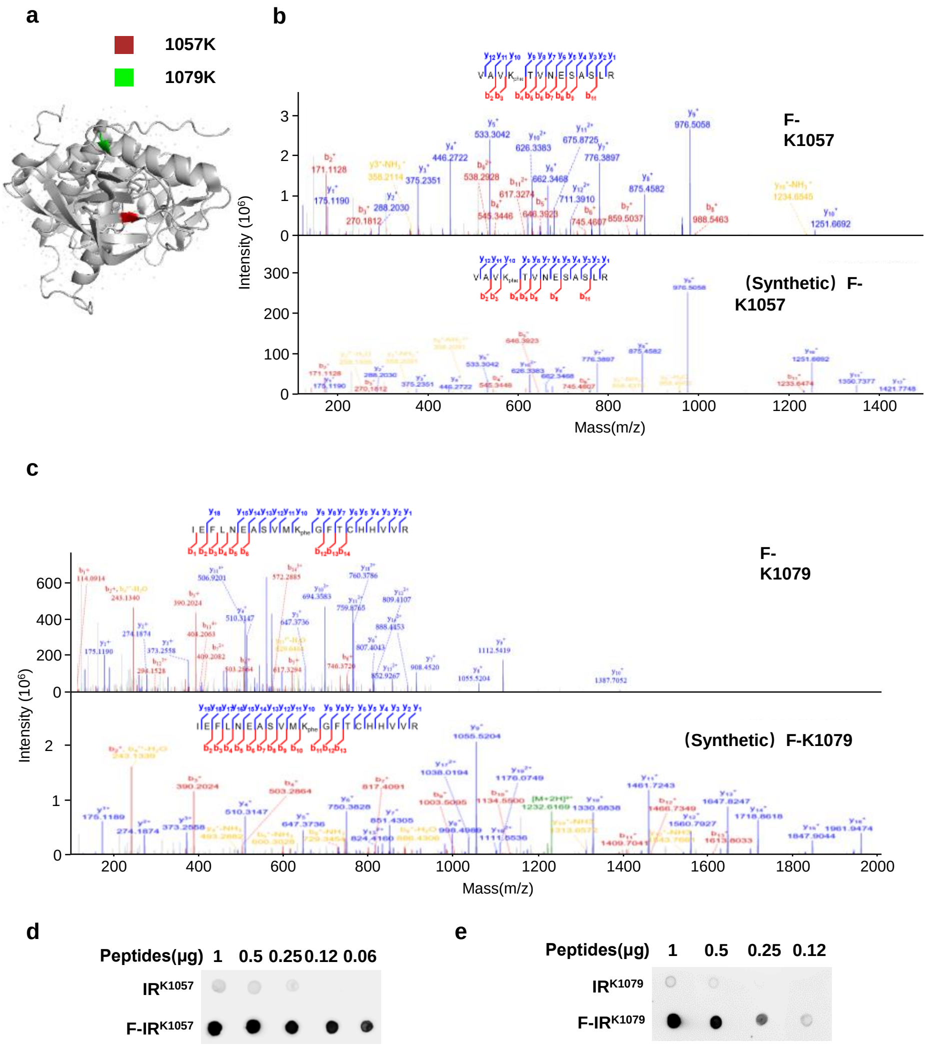

Supplementary Fig. 5, FARS phenylalanylated Lys1079 and Lys1057 of IR, related to Fig. 3.

a, K1057 and K1079 are on the surface of IRβ. PyMol simulation showed that K1057 (red) and K1079 (green) are located at the surface of the IRβ protein (PDB: 5hhw).

b-c, **MS identification and confirmation of F-K1057 and F-K1079**. The MS/MS spectra that identified K1057 (**b**) and K1079 (**c**) were phenylalanylated and matched the MS/MS spectra from synthetic F-K1057 and F-K1079 peptides (lower, **b** and **c**).

d-e, **Characterization of site-specific F-K1057 and F-K1079 antibodies**. F-K1057 (**d**) and F-K1079 (**e**) site-specific antibodies were tested for their reactivity to F-K1057- and F-K1079-containing peptides (TRVAVK$_{1057phe}$TVNES and EASVMK$_{1079phe}$GFTCH) and peptides with the same amino acid sequences devoid of phenylalanylated lysine.

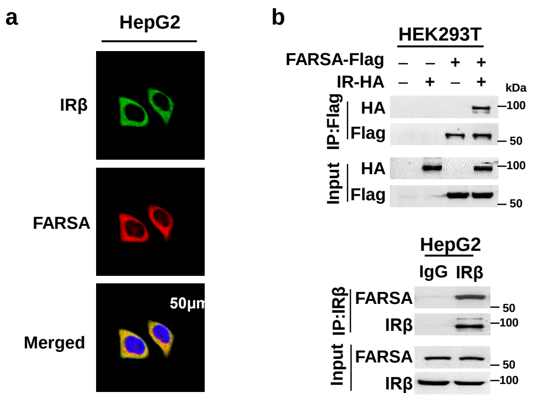

Supplementary Fig. 6, FARSA interacted with IR, related to Fig. 3.

a, FARSA colocalized with IRβ. Immunofluorescence staining of IRβ (green) and FARSA (red) in HepG2 cells is shown.

b, **FARSA interacted with IRβ**. A coimmunoprecipitation assay showed that ectopically expressed (upper) and endogenous (lower) FARSA interacted with IRβ in HEK293T and HepG2 cells.

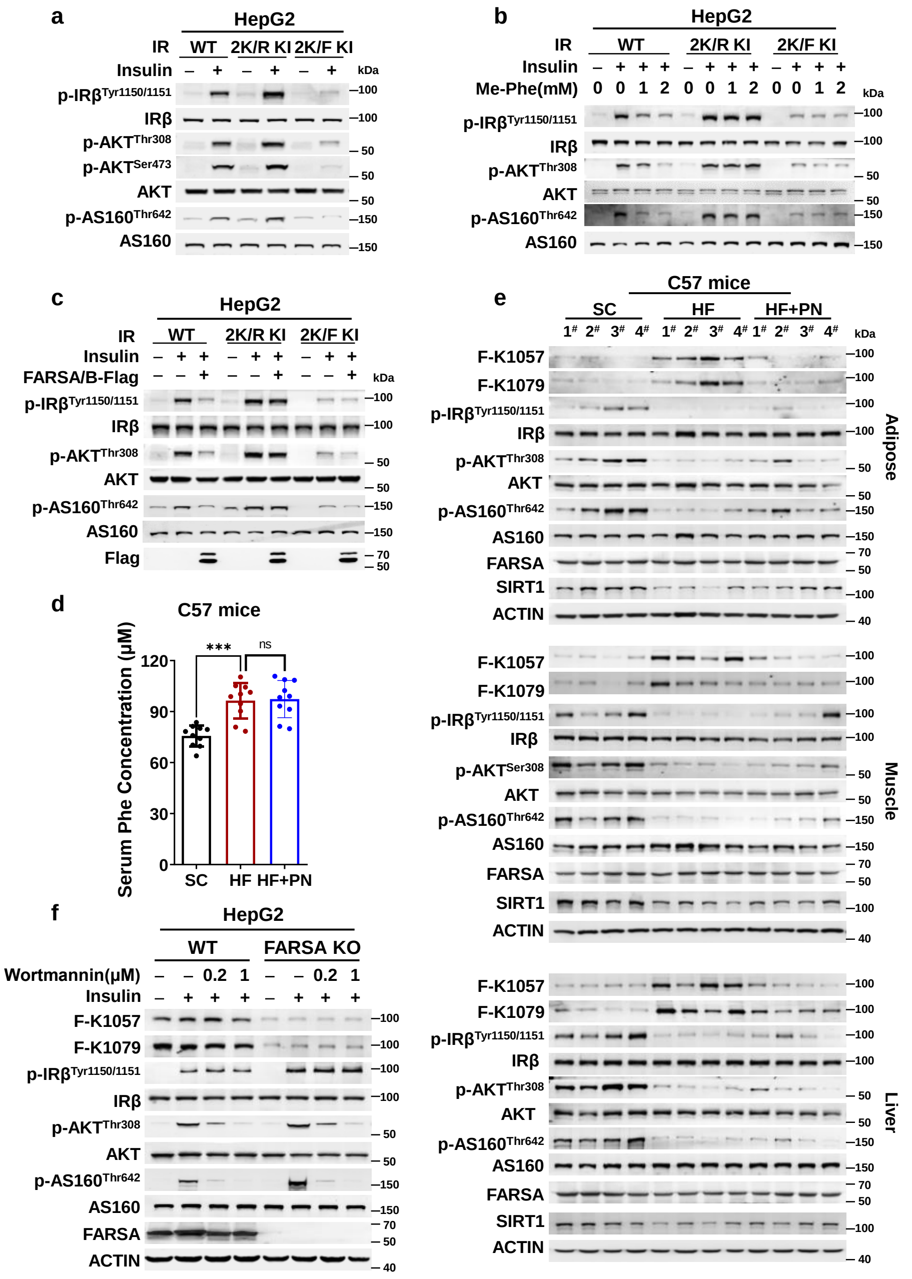

Supplementary Fig. 7, F-K1057/1079 inactivated insulin signaling, related to Fig. 4.

a, K1057/1079 phenylalanylation inactivated IRβ. The effects of insulin on the phosphorylation levels of components of the insulin signaling pathway were detected in IRβ, IRβ$^{2K/R}$ and IRβ$^{2K/F}$ knock-in HepG2 cells.

b, **Absence of K1057/K1079 abrogated Me-Phe to alter insulin signaling.** The responses of insulin signaling to Me-Phe treatment were detected in IRβ, IRβ$^{2K/R}$ and IRβ$^{2K/F}$ knock-in HepG2 cells.

c, FARS overexpression failed to inhibit insulin signaling in HepG2 cells lack of K1057/K1079. The responses of insulin signaling to wild type and mutant FARSA/B overexpression were detected in IRβ, IRβ$^{2K/R}$ and IRβ$^{2K/F}$ knock-in HepG2 cells.

d-e, High fat diet increased F-K1057 and F-K1079 levels and inhibited insulin signaling. Male C57 mice were fed high fat diet or phenylalaninol-supplemented chow for 12 weeks (n = 10). The serum Phe levels (**d**), F-K1057/F-K1079 levels and phosphorylation of components of the insulin signaling pathway (**e**) were detected in adipose, muscle and liver tissues. Significance was indicated as two-tailed, unpaired, t test. Values are expressed as the mean ± SEM. *p < 0.05, **p < 0.01, ***p < 0.001.

f, IRβ phenylalanylation is sufficient but not required for insulin signaling inactivation. F-K1057/1079 levels and insulin signaling were detected in wild type and FARSA KO HepG2 cells that were either untreated or treated with wortmannin.

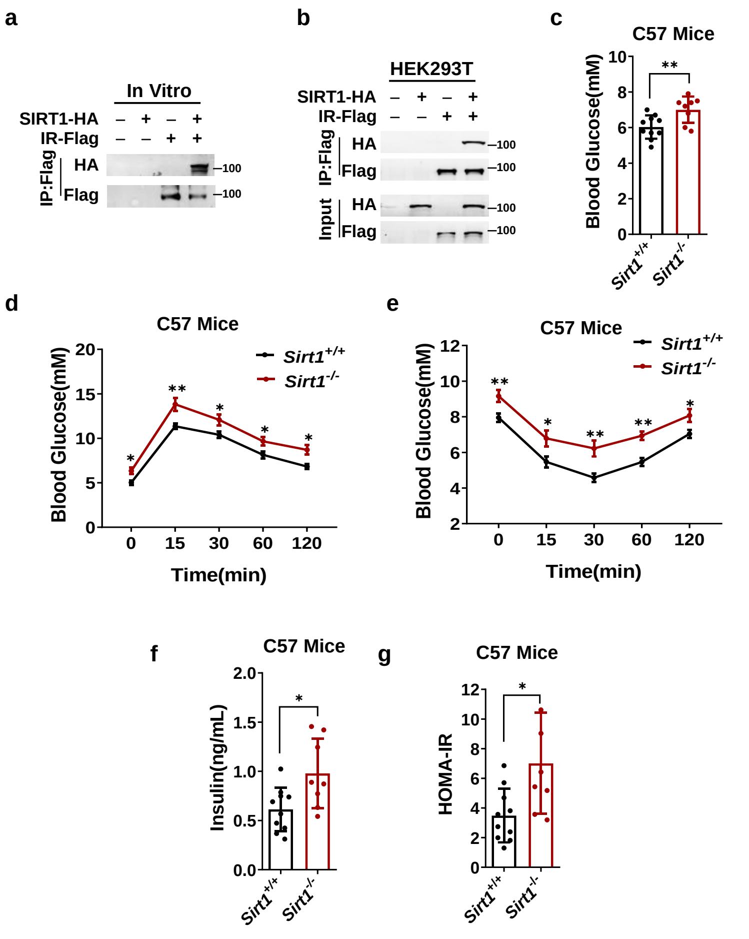

Supplementary Fig. 8, SIRT1 removed F-K1057/1079 and sensitized insulin signaling, related to Fig. 5.

a-b, **SIRT1 interacted with IRβ**. Recombinant SIRT1 pulled down IRβ (**a**) and ectopically expressed SIRT1 coimmunoprecipitated with overexpressed IRβ in HEK293T cells (**b**).

c-g, Sirt1 knockout induced T2D symptoms. Blood glucose levels (**c**), glucose tolerance tests (**d**), insulin tolerance tests (**e**), serum insulin levels (**f**), and HOMA-IR values (**g**) of fasted wild-type (n = 10) and *Sirt1*$^{-/-}$ (n = 8) C57 mice were measured.

Student's t-tests (unpaired, two-tailed) are applied for all statistical analyses in this figure. Values are expressed as the mean ± SEM. Significance was indicated as *p < 0.05, **p < 0.01, ***p < 0.001.

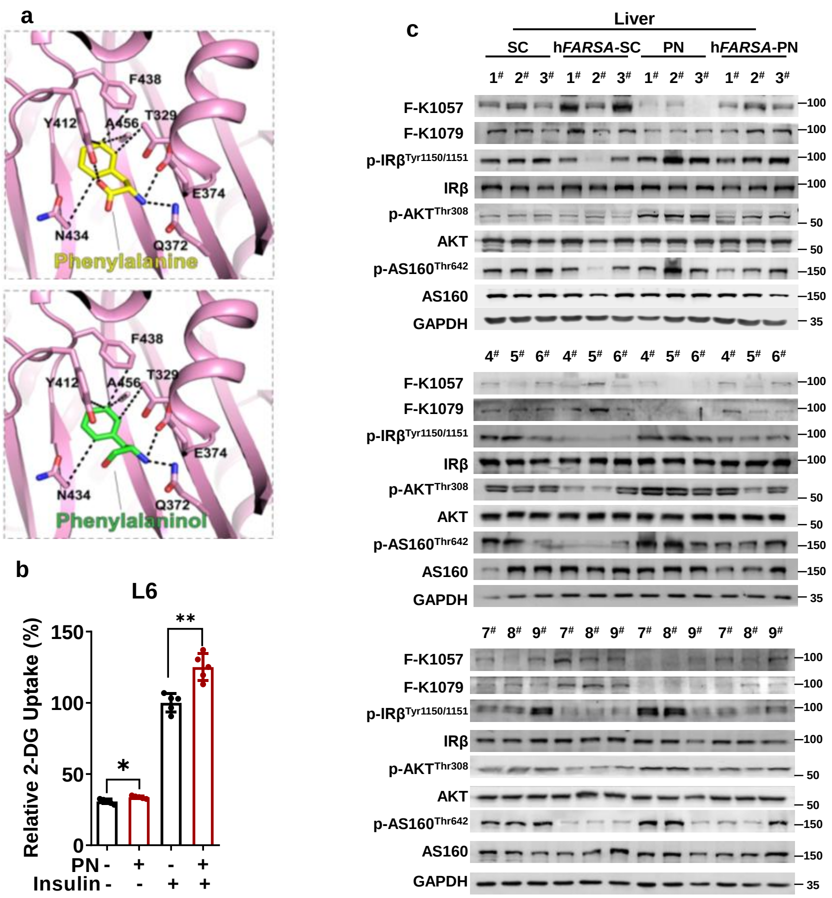

Supplementary Fig. 9, Decreasing F-K1057/1079 restored insulin signaling, related to Fig. 7.

a, **Phenylalaninol occupied the phenylalanine binding pocket of FARSA**. Projected detailed interactions between phenylalanine, phenylalaninol and FARSA were illustrated.

b, **Phenylalaninol treatment improved glucose uptake by L6 myocytes**. The effects of phenylalaninol on 2-DG uptake by L6 cells were measured in the absence and presence of insulin treatments (n = 5). Significance was indicated as two-tailed, unpaired, t test. Values are expressed as the mean ± SEM. *$p < 0.05$, **$p < 0.01$, ***$p < 0.001$.

c, Phenylalaninol decreased F-K1057/1079 levels and relieved diabetic symptoms in *hFARSA*-transgenic mice. Original western blot results of **Fig. 7e**.

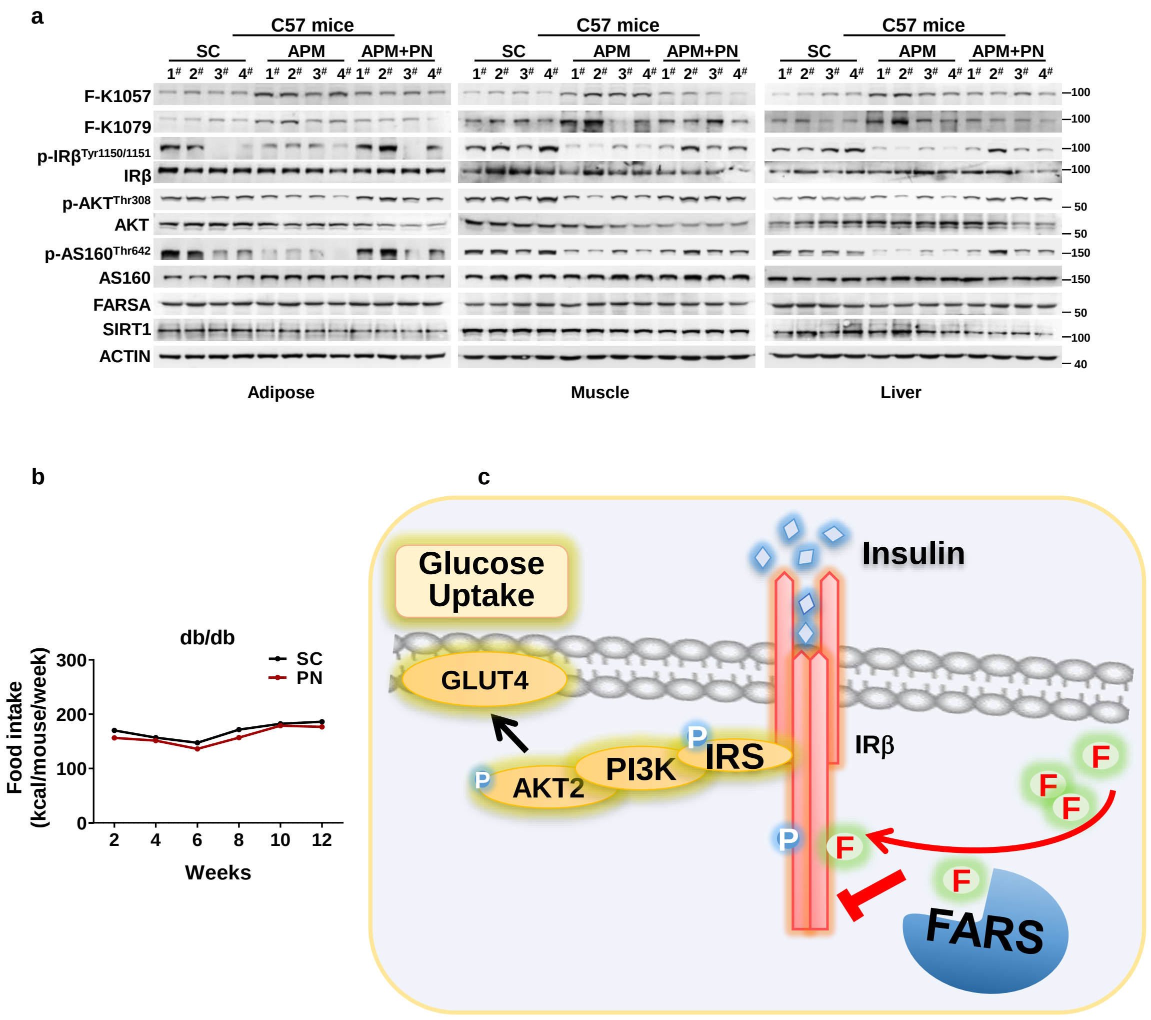

Supplementary Fig. 10, Inhibiting F-K1057/1079 restored insulin signaling, related to Fig. 7

a, **PN decreases Aspartame-increased F-K1057 /1079 levels and inhibited insulin signaling**. Male C57 mice were fed aspartame chow or aspartame/phenylalaninol-supplemented chow for 12 weeks (n = 10). The F-K1057/1079 levels, phosphorylation levels of components of the insulin signaling pathway, FARSA and SIRT1 protein levels were detected in adipose, muscle and liver tissues.

b, Phenylalaninol did not affect the food intake of db/db mice. Food intake of db/db mice fed standard chow (n=10) and phenylalaninol-supplemented chow (n=10) was monitored over time.

c, Schematic illustration of insulin receptor and signaling regulation by phenylalanylation. Cytosolic phenylalanine (F) modifies IRβ via catalyzation by FARS, which inactivates insulin signaling and inhibits glucose uptake.

Characteristics of control and T2D patients

Characteristic	Con(n=60)	T2D(n=62)	p value
Sex(M/F)	33/27	35/27	0.873
Age(years)	51.7±10.9	54.2±9.3	0.179
Weight(Kg)	71.4±14.0	75.4±17.1	0.157
Height(cm)	165.6±8.4	166.2±8.6	0.692
Fasting Blood Glucose(mM)	5.4±0.9	8.5±2.8	4.7×10^{-12}
Insulin(mU/L)	7.8±3.9	18.9±32.2	0.009
HbA1c(%)	5.7±0.5	8.8±2.0	6.3×10^{-18}
Triglyceride(mM)	1.9±1.5	2.1±1.7	0.498
Cholesterol(mM)	4.7±1.3	4.4±1.4	0.292
LDL Cholesterol(mM)	2.9±1.0	2.9±1.0	0.860
HDL Cholesterol(mM)	1.1±0.3	1.1±0.2	0.496

Supplementary Table 1, Clinical information of T2D patients and health subjects used in this study.

Student's t-tests (unpaired, two-tailed) are applied for all statistical analyses in this table. Values are expressed as the mean ± SEM.
