# Supplementary material for: Phenylalanine impairs insulin signaling and inhibits glucose uptake through modification of IRβ
Source: Nat Commun. 2022 Jul 25;13:4291. doi: 10.1038/s41467-022-32000-0 (PMC9314339; doi:10.1038/s41467-022-32000-0)
Supplement: Supplementary file 3 — Reporting Summary [file 41467_2022_32000_MOESM3_ESM.pdf]

nature portfolio

Corresponding author(s): Shimin Zhao

Last updated by author(s): Jun 9, 2022

Reporting Summary

Nature Portfolio wishes to improve the reproducibility of the work that we publish. This form provides structure for consistency and transparency in reporting. For further information on Nature Portfolio policies, see our Editorial Policies and the Editorial Policy Checklist.

Statistics

For all statistical analyses, confirm that the following items are present in the figure legend, table legend, main text, or Methods section.

n/a	Confirmed	
☐	☒	The exact sample size (*n*) for each experimental group/condition, given as a discrete number and unit of measurement
☐	☒	A statement on whether measurements were taken from distinct samples or whether the same sample was measured repeatedly
☐	☒	The statistical test(s) used AND whether they are one- or two-sided<br>*Only common tests should be described solely by name; describe more complex techniques in the Methods section.*
☒	☐	A description of all covariates tested
☒	☐	A description of any assumptions or corrections, such as tests of normality and adjustment for multiple comparisons
☐	☒	A full description of the statistical parameters including central tendency (e.g. means) or other basic estimates (e.g. regression coefficient) AND variation (e.g. standard deviation) or associated estimates of uncertainty (e.g. confidence intervals)
☐	☒	For null hypothesis testing, the test statistic (e.g. *F*, *t*, *r*) with confidence intervals, effect sizes, degrees of freedom and *P* value noted<br>*Give P values as exact values whenever suitable.*
☒	☐	For Bayesian analysis, information on the choice of priors and Markov chain Monte Carlo settings
☒	☐	For hierarchical and complex designs, identification of the appropriate level for tests and full reporting of outcomes
☒	☐	Estimates of effect sizes (e.g. Cohen's *d*, Pearson's *r*), indicating how they were calculated

Our web collection on statistics for biologists contains articles on many of the points above.

Software and code

Policy information about availability of computer code

Data collection	Western blot film and gel scan: Typhoon FLA 9500 (GE Healthcare, Little Chalfont, UK);<br>Confocal: Zeiss LSM 710.
Data analysis	Curve fitting and statistical analysis:GraphPad 8.0 ;<br>Image quantification and analysis: Image J 1.8.0.

For manuscripts utilizing custom algorithms or software that are central to the research but not yet described in published literature, software must be made available to editors and reviewers. We strongly encourage code deposition in a community repository (e.g. GitHub). See the Nature Portfolio guidelines for submitting code & software for further information.

Data

Policy information about availability of data

All manuscripts must include a data availability statement. This statement should provide the following information, where applicable:

- Accession codes, unique identifiers, or web links for publicly available datasets
- A description of any restrictions on data availability
- For clinical datasets or third party data, please ensure that the statement adheres to our policy

Raw data of Figures 1-7 and Supplementary Figure 1-10 are available.

Field-specific reporting

Please select the one below that is the best fit for your research. If you are not sure, read the appropriate sections before making your selection.

☒ Life sciences ☐ Behavioural & social sciences ☐ Ecological, evolutionary & environmental sciences

For a reference copy of the document with all sections, see nature.com/documents/nr-reporting-summary-flat.pdf

Life sciences study design

All studies must disclose on these points even when the disclosure is negative.

Sample size	Sample size was indicated in the figure legends or in the text. For all the measurements, three or more repeats were performed in SEM variation test were performed and indicated in the manuscript. To enhance reproducibility, no less than five animals were assigned per group. Animal experimental numbers were determined by previous studies and our experience to reveal statistical significance(Axelsson et al., 1996; Bauer et al., 2010; Carlsson et al., 2013; Ito et al., 2008; Zhou et al., 2018). No statistical method was used to predetermine sample size.
Data exclusions	No reported data was excluded.
Replication	Biological replicates (3-6) were indicated in the figure legend. Western blots were repeated at least twice and other measurements were repeated at least three times.
Randomization	Allocation was random.
Blinding	Investigators were blinded to group allocation.

Reporting for specific materials, systems and methods

We require information from authors about some types of materials, experimental systems and methods used in many studies. Here, indicate whether each material, system or method listed is relevant to your study. If you are not sure if a list item applies to your research, read the appropriate section before selecting a response.

Materials & experimental systems

n/a	Involved in the study	
☐	☒	Antibodies
☐	☒	Eukaryotic cell lines
☒	☐	Palaeontology and archaeology
☐	☒	Animals and other organisms
☐	☒	Human research participants
☒	☐	Clinical data
☐	☒	Dual use research of concern

Methods

n/a	Involved in the study	
☒	☐	ChIP-seq
☒	☐	Flow cytometry
☒	☐	MRI-based neuroimaging

Antibodies

Antibodies used

IRβ⍰Rabbit? Cell Signaling Technology Cat#3025 1:1000
IRβ⍰Mouse? Cell Signaling Technology Cat#3020 1:500
p-IRβTyr1150/1151 Cell Signaling Technology Cat#3024 1:1000
IRS1 Cell Signaling Technology Cat#2382 1:1000
pan-phospho-Tyrosine Cell Signaling Technology Cat#9461 1:2000
AKT Cell Signaling Technology Cat#4685 1:3000
p-AKTThr308 Cell Signaling Technology Cat#13038 1:1000
p-AKTSer473 Cell Signaling Technology Cat#4060 1:3000
AS160 Cell Signaling Technology Cat#2670 1:1000
p-AS160Thr462 Cell Signaling Technology Cat#8881 1:1000
SHC Cell Signaling Technology Cat# 2432 1:1000
p-SHCTyr239/240 Cell Signaling Technologym Cat# 2434 1:1000
ERK1/2 Cell Signaling Technology Cat# 9102 1:1000
p-ERK1/2Tyr202/204 Cell Signaling Technology Cat# 9101 1:1000
S6K Cell aling Technology Cat#2708 1:1000
p-S6KThr389 Cell Signaling Technology Cat#9234 1:1000
GSK3β Cell Signaling Technology Cat#12456 1:1000
p-GSK3βSer9 Cell Signaling Technology Cat#5558 1:1000
SIRT1 (Rabbit) Cell Signaling Technology Cat#9475 1:1000
SIRT1 (Rabbit) Proteintech Cat#13161-1-AP 1:1000
SIRT1 (Mouse) Proteintech Cat#60303-1-lg 1:1000

FARSA (Rabbit) Proteintech Cat#18121-1-AP 1:1000
FARSA (Mouse) Santacruz Cat#sc-100987 1:500
FARS2 Proteintech Cat#16436-1-AP 1:1000
PAH Abcam Cat#ab178430 1:3000
IRβ?Rabbit? Abcam Cat#ab69508 1:1000
GLUT4 Abcam Cat#ab654 1:3000
F-IRK1057 Home-made N/A 1:500
F-IRK1079 Home-made N/A 1:500
Flag Abmart Cat#M20008 1:3000
HA Abmart Cat#M20003 1:3000
Myc Abmart Cat# M20002 1:3000
Actin GenScript Cat#A00702-100 1:5000
anti-rabbit secondary antibodies GenScript Cat#A00098 1:5000
anti-mouse secondary antibodies GenScript Cat#A00160 1:5000
Alexa Fluor Plus 488 donkey anti-mouse IgG secondary antibody Invitrogen Cat#A32766TR 1:5000
Alexa Fluor Plus 555 donkey anti-rabbit IgG secondary antibody Invitrogen Cat#A32794 1:5000
Alexa Fluor Plus 488 goat anti-rabbit IgG secondary antibody Invitrogen Cat#A11008 1:5000

Validation

IRβ?Rabbit? Cell Signaling Technology Cat#3025 https://www.cellsignal.cn/products/primary-antibodies/insulin-receptor-b-4b8-rabbit-mab/3025?site-search-type=Products&N=4294956287&Ntt=3025&fromPage=plp&_requestid=4515014
IRβ?Mouse? Cell Signaling Technology Cat#3020 https://www.cellsignal.cn/products/primary-antibodies/insulin-receptor-b-l55b10-mouse-mab/3020?site-search-type=Products&N=4294956287&Ntt=3020&fromPage=plp&_requestid=4515161
p-IRβTyr1150/1151 Cell Signaling Technology Cat#3024 https://www.cellsignal.cn/products/primary-antibodies/phospho-igf-i-receptor-b-tyr1135-1136-insulin-receptor-b-tyr1150-1151-19h7-rabbit-mab/3024?site-search-type=Products&N=4294956287&Ntt=3024&fromPage=plp&_requestid=4515487
IRS1 Cell Signaling Technology Cat#2382 https://www.cellsignal.cn/products/primary-antibodies/irs-1-antibody/2382?site-search-type=Products&N=4294956287&Ntt=2382&fromPage=plp&_requestid=4515826
pan-phospho-Tyrosine Cell Signaling Technology Cat#9411 https://www.cellsignal.cn/products/primary-antibodies/phospho-tyrosine-mouse-mab-p-tyr-100/9411?site-search-type=Products&N=4294956287&Ntt=phospho-tyrosine&fromPage=plp
AKT Cell Signaling Technology Cat#4685 https://www.cellsignal.cn/products/primary-antibodies/akt-pan-11e7-rabbit-mab/4685?site-search-type=Products&N=4294956287&Ntt=4685&fromPage=plp&_requestid=4516023
p-AKTThr308 Cell Signaling Technology Cat#13038 https://www.cellsignal.cn/products/primary-antibodies/phospho-akt-thr308-d25e6-xp-rabbit-mab/13038?site-search-type=Products&N=4294956287&Ntt=13038&fromPage=plp&_requestid=4516241
p-AKTSer473 Cell Signaling Technology Cat#4060 https://www.cellsignal.cn/products/primary-antibodies/phospho-akt-ser473-d9e-xp-rabbit-mab/4060?site-search-type=Products&N=4294956287&Ntt=4060&fromPage=plp&_requestid=4516295
AS160 Cell Signaling Technology Cat#2670 https://www.cellsignal.cn/products/primary-antibodies/as160-c69a7-rabbit-mab/2670?site-search-type=Products&N=4294956287&Ntt=2670&fromPage=plp&_requestid=4516355
p-AS160Thr462 Cell Signaling Technology Cat#8881 https://www.cellsignal.cn/products/primary-antibodies/phospho-as160-thr642-d27e6-rabbit-mab/8881?site-search-type=Products&N=4294956287&Ntt=8881&fromPage=plp&_requestid=4516390
SHC Cell Signaling Technology Cat# 2432 https://www.cellsignal.cn/products/primary-antibodies/shc-antibody/2432?site-search-type=Products&N=4294956287&Ntt=2432&fromPage=plp&_requestid=4516461
p-SHCTyr239/240 Cell Signaling Technologym Cat# 2434 https://www.cellsignal.cn/products/primary-antibodies/phospho-shc-tyr239-240-antibody/2434?site-search-type=Products&N=4294956287&Ntt=2434&fromPage=plp&_requestid=4516522
ERK1/2 Cell Signaling Technology Cat# 9102 https://www.cellsignal.cn/products/primary-antibodies/p44-42-mapk-erk1-2-antibody/9102?site-search-type=Products&N=4294956287&Ntt=9102&fromPage=plp&_requestid=4516555
p-ERK1/2Tyr202/204 Cell Signaling Technology Cat# 9101 https://www.cellsignal.cn/products/primary-antibodies/phospho-p44-42-mapk-erk1-2-thr202-tyr204-antibody/9101?site-search-type=Products&N=4294956287&Ntt=9101&fromPage=plp&_requestid=4516587
S6K Cell aling Technology Cat#2708 https://www.cellsignal.cn/products/primary-antibodies/p70-s6-kinase-49d7-rabbit-mab/2708?site-search-type=Products&N=4294956287&Ntt=2708&fromPage=plp
p-S6KThr389 Cell Signaling Technology Cat#9234 https://www.cellsignal.cn/products/primary-antibodies/phospho-p70-s6-kinase-thr389-108d2-rabbit-mab/9234?site-search-type=Products&N=4294956287&Ntt=9234&fromPage=plp
GSK3β Cell Signaling Technology Cat#12456 https://www.cellsignal.cn/products/primary-antibodies/gsk-3b-d5c5z-xp-rabbit-mab/12456?site-search-type=Products&N=4294956287&Ntt=12456&fromPage=plp&_requestid=4516723
p-GSK3βSer9 Cell Signaling Technology Cat#5558 https://www.cellsignal.cn/products/primary-antibodies/phospho-gsk-3b-ser9-d85e12-xp-rabbit-mab/5558?site-search-type=Products&N=4294956287&Ntt=5558&fromPage=plp&_requestid=4516768
SIRT1 (Rabbit) Cell Signaling Technology Cat#9475 https://www.cellsignal.cn/products/primary-antibodies/sirt1-d1d7-rabbit-mab/9475?site-search-type=Products&N=4294956287&Ntt=9475&fromPage=plp&_requestid=4516803
SIRT1 (Rabbit) Proteintech Cat#13161-1-AP https://www.ptgcn.com/products/SIRT1-Antibody-13161-1-AP.htm
SIRT1 (Mouse) Proteintech Cat#60303-1-lg https://www.ptgcn.com/products/SIRT1-Antibody-60303-1-Ig.htm
FARSA (Rabbit) Proteintech Cat#18121-1-AP https://www.ptgcn.com/products/FARSA-Antibody-18121-1-AP.htm
FARSA (Mouse) Santacruz Cat#sc-100987 https://www.scbt.com/p/farsla-antibody-l-8?requestFrom=search
FARS2 Proteintech Cat#16436-1-AP https://www.ptgcn.com/products/FARS2-Antibody-16436-1-AP.htm
PAH Abcam Cat#ab178430 https://www.abcam.cn/pah-antibody-epr12380-ab178430.html
IRβ?Rabbit? Abcam Cat#ab69508 https://www.abcam.cn/insulin-receptor-beta-antibody-c18c4-ab69508.html
GLUT4 Abcam Cat#ab654 https://www.abcam.cn/glucose-transporter-glut4-antibody-ab654.html
Flag Abmart Cat#M20008 http://www.ab-mart.com.cn/page.aspx?node=%2060%20&id=%20968
HA Abmart Cat#M20003 http://www.ab-mart.com.cn/page.aspx?node=%2060%20&id=%20963
Myc Abmart Cat# M20002 http://www.ab-mart.com.cn/page.aspx?node=%2060%20&id=%20962
Actin GenScript Cat#A00702-100 https://www.genscript.com/search?q=A00702-100&search=Search
anti-rabbit secondary antibodies GenScript Cat#A00098 https://www.genscript.com/antibody/A00098-Goat_Anti_Rabbit_IgG_Antibody_H_L_HRP_pAb.html?page_no=1&position_no=1&sensors=googlesearch
anti-mouse secondary antibodies GenScript Cat#A00160 https://www.genscript.com/antibody/A00160-

Goat_Anti_Mouse_IgG_Antibody_H_L_HRP_pAb_.html?page_no=1&position_no=1&sensors=googlesearch
Alexa Fluor Plus 488 donkey anti-mouse IgG secondary antibody Invitrogen Cat#A32766TR https://www.thermofisher.cn/cn/zh/antibody/product/Donkey-anti-Mouse-IgG-H-L-Highly-Cross-Adsorbed-Secondary-Antibody-Polyclonal/A32766TR
Alexa Fluor Plus 555 donkey anti-rabbit IgG secondary antibody Invitrogen Cat#A32794 https://www.thermofisher.cn/cn/zh/antibody/product/Donkey-anti-Rabbit-IgG-H-L-Highly-Cross-Adsorbed-Secondary-Antibody-Polyclonal/A32794
Alexa Fluor Plus 488 goat anti-rabbit IgG secondary antibody Invitrogen Cat#A11008 https://www.thermofisher.cn/cn/zh/antibody/product/Goat-anti-Rabbit-IgG-H-L-Cross-Adsorbed-Secondary-Antibody-Polyclonal/A-11008

The Anti-F-K1057 (1:500) and Anti-F-K1079 (1:500) antibodies were generated by Abmart Shanghai Co., Ltd during this study. Briefly, synthetic peptides (KGEAETRVAVK1057PheTVNESASLRE) and IEFLNEASVMK1079PheGFTCHHVVR) were conjugated to keyhole limpet hemocyanin (KLH) as antigen. Rabbits were immunized by four doses of subcutaneous injection at two weeks interval between each injection before the rabbits were sacrificed for anti-sera. Antibodies were immunoaffinity purified by antigen and tested for specificities by blot assay before employed for western blotting analysis. Validation results were included in the manuscript as Figure S5d-S5e.

Eukaryotic cell lines

Policy information about cell lines

Cell line source(s)	HEK293T cells (human ATCC Number: CRL-11268)<br>HepG2 (Human ATCC Number: HB-8065)<br>3T3-L1 adipocytes (Mouse Stem Cell Bank, Chinese Academy of Sciences Number: SCSP-5038)<br>L6 rat skeletal myoblasts (Rat Stem Cell Bank, Chinese Academy of Sciences Number:GNR 4)
Authentication	All cell lines used in this study was authenticated by: morphology check by microscope, and identity verification with STR analysis (DNA fingerprinting).
Mycoplasma contamination	All cell lines were tested negative for mycoplasma contaminition
Commonly misidentified lines (See ICLAC register)	No commonly misidentified cell lines were used in the study.

Animals and other organisms

Policy information about studies involving animals; ARRIVE guidelines recommended for reporting animal research

Laboratory animals	Male C57BL/6J mice, male db/db mice, and the hFARSA transgenic mice generated in a C57BL/6 genetic background of 4-20 weeks were used for this study. All mice were housed in specific pathogen-free conditions at room temperature, 40-60% humidity, on 14h light/10h dark cycles.
Wild animals	The study did not involve wild animals .
Field-collected samples	The study did not involve samples collected from the field.
Ethics oversight	All animal procedures were in accordance with the animal care committee at Fudan University.

Note that full information on the approval of the study protocol must also be provided in the manuscript.

Human research participants

Policy information about studies involving human research participants

Population characteristics	T2D patients and matched healthy subjects were obtained from volunteers of Huashan hospital, Shanghai with known age, height, fasting blood glucose, insulin, HbAc1, triglyceride, cholesterol, LDL cholesterol, HDL cholesterol and type 2 diabetes status. The mean age (years) for healthy and T2D participants were 51.7±10.9 and 54.2±9.3 respectively. The gender (Male/female) for healthy and T2D participants were 33/27 and 35/27 respectively. The baseline characteristics of all participants are shown in Supplementary Table 1.
Recruitment	In this study, all participants were recruited from Endocrinology department, Huashan hospitals affliated to Fudan university in Shanghai, People's Republic of China, since 2020. nclusion criteria included willingness to participate in the study, age≥18 years. Exclusion criteria included pregnancy and major psychiatric disorders.
Ethics oversight	The study was approved by the Human Investigation Ethics Committee of Huashan Hospital (KY2019-404). With a full understanding of the study, each participant signed the informed consent form voluntarily.

Note that full information on the approval of the study protocol must also be provided in the manuscript.

Dual use research of concern

Policy information about dual use research of concern

Hazards

Could the accidental, deliberate or reckless misuse of agents or technologies generated in the work, or the application of information presented in the manuscript, pose a threat to:

No	Yes	
☒	☐	Public health
☒	☐	National security
☒	☐	Crops and/or livestock
☒	☐	Ecosystems
☒	☐	Any other significant area

Experiments of concern

Does the work involve any of these experiments of concern:

No	Yes	
☒	☐	Demonstrate how to render a vaccine ineffective
☒	☐	Confer resistance to therapeutically useful antibiotics or antiviral agents
☒	☐	Enhance the virulence of a pathogen or render a nonpathogen virulent
☒	☐	Increase transmissibility of a pathogen
☒	☐	Alter the host range of a pathogen
☒	☐	Enable evasion of diagnostic/detection modalities
☒	☐	Enable the weaponization of a biological agent or toxin
☒	☐	Any other potentially harmful combination of experiments and agents
